# Development of Bactericidal Peptides against Multidrug-Resistant *Acinetobacter baumannii* with Enhanced Stability and Low Toxicity

**DOI:** 10.3390/ijms23042191

**Published:** 2022-02-16

**Authors:** Prakash Kishore Hazam, Chin-Cheng Cheng, Chu-Yi Hsieh, Wen-Chun Lin, Po-Hsien Hsu, Te-Li Chen, Yi-Tzu Lee, Jyh-Yih Chen

**Affiliations:** 1 Marine Research Station, Institute of Cellular and Organismic Biology, Academia Sinica, 23-10 Dahuen Road., Ilan 262, Taiwan; prakashkishor.hazam@gmail.com (P.K.H.); joy.cde49@gmail.com (C.-Y.H.); hagirl19@gmail.com (W.-C.L.); 2 Institute of Fisheries Science, National Taiwan University, 1 Roosevelt Road, Section 4, Taipei 112, Taiwan; eddie040886@gmail.com (C.-C.C.); ericchu102001@gmail.com (P.-H.H.); 3 Graduate Institute of Life Sciences, National Defense Medical Center, Taipei 112, Taiwan; tecklayyy@gmail.com; 4 Institute of Clinical Medicine, School of Medicine, National Yang-Ming University, Taipei 112, Taiwan; 5 Department of Emergency Medicine, Taipei Veterans General Hospital, No. 201, Section 2, Shipai Road, Taipei 112, Taiwan; s851009@yahoo.com.tw; 6 Faculty of Medicine, School of Medicine, National Yang-Ming University, No. 155, Section 2, Linong Street, Taipei 112, Taiwan; 7 Rong Hsing Research Center for Translational Medicine, National Chung Hsing University, Taichung 402, Taiwan; 8 The iEGG and Animal Biotechnology Center, National Chung Hsing University, Taichung 402, Taiwan

**Keywords:** antimicrobial resistance, tilapia piscidin, antimicrobial peptide, MDR infection

## Abstract

Pathogenic superbugs are the root cause of untreatable complex infections with limited or no treatment options. These infections are becoming more common as clinical antibiotics have lost their effectiveness over time. Therefore, the development of novel antibacterial agents is urgently needed to counter these microbes. Antimicrobial peptides (AMPs) are a viable treatment option due to their bactericidal potency against multiple microbial classes. AMPs are naturally selected physiological microbicidal agents that are found in all forms of organisms. In the present study, we developed two tilapia piscidin 2 (TP2)-based AMPs for antimicrobial application. Unlike the parent peptide, the redesigned peptides showed significant antimicrobial activity against multidrug-resistant bacterial species. These peptides also showed minimal cytotoxicity. In addition, they were significantly active in the presence of physiological salts, 50% human serum and elevated temperature. The designed peptides also showed synergistic activity when combined with clinical antibiotics. The current approach demonstrates a fruitful strategy for developing potential AMPs for antimicrobial application. Such AMPs have potential for progression to further trials and drug development investigations.

## 1. Introduction

Antibiotics are primary agents used for neutralizing pathogenic infections. Since their discovery, they have been an integral part of clinical therapy [1]. However, there is a clear indication of a significant reduction in their activity due to multiple factors [2]. One of the key factors resulting in reduced antimicrobial potential is the recurring presence of multidrug-resistant (MDR) bacterial species. Researchers have estimated that 10 million annual deaths could occur due to microbial infection if we fail to develop effective antimicrobials [3]. The World Health Organization (WHO) has categorized critical, high-priority and medium-priority pathogens according to the need for new therapies to treat them. The critical priority group comprises *Acinetobacter baumannii* (carbapenem-resistant) and *Pseudomonas aeruginosa*. The high-priority group contains *Staphylococcus aureus* (methicillin-resistant) and *Helicobacter pylori* (clarithromycin-resistant) and the medium-priority group consists of *Haemophilus influenzae* (ampicillin-resistant) [4]. Therefore, the search for new antibiotics and potential alternatives is among the primary interests of the health care system [1]. To this end, antimicrobial peptides have been shown to be potential antibiotics owing to their broad-spectrum activity against multidrug-resistant bacteria [5]. Antimicrobial peptides (AMPs) are defense molecules found in all organisms [6]. They are mostly amphipathic with a net positive charge. AMPs act through membrane rupturing mechanisms. AMPs primarily interact through electrostatic interactions between the anionic bacterial membrane and the cationic peptide, causing degradation of the bacterial membrane [5].

In the current study, we used a pair of peptides known as TP2-5 and TP2-6 to develop potential bactericidal agents against multidrug-resistant *A. baumannii* [7]. The peptide pair was developed from tilapia piscidin 2 (TP2), an inactive antibacterial peptide found in *Oreochromis niloticus* (Nile tilapia) [8]. TP2-5 and TP2-6 are modified variants that have improved cationicity and amphipathic balance, which resulted in significant improvement in their antimicrobial potential in normal media [7]. Therefore, we selected this pair to assess their antimicrobial potential in the presence of human serum, lung surfactant, physiological ions and elevated temperature. Additionally, we screened the peptides against a series of MDR *A. baumannii* bacterial species (Supplementary Table S1). The peptides significantly retained their activity under the test conditions and against the MDR bacterial species. Additionally, the peptides were nontoxic and possessed antibiofilm potential. Both peptides showed synergistic potential against MDR bacterial species when combined with meropenem. These peptides retained significant activity under physiological conditions with potential activity against clinically important MDR *A. baumannii* bacterial species.

## 2. Results and Discussion

### 2.1. Peptide Design

A pair of test peptides, TP2-5 and TP2-6, were selected from a previous design based on tilapia piscidin 2 (TP2) [7]. Tilapia piscidin 2 is a peptide without antimicrobial activity obtained from *Oreochromis niloticus* [9]. The original peptide TP2 (GECIWDAIFHGAKHFLHRLVNP) possesses negligible or no net positive charge [8]. However, peptides TP2-5 (KKCIAKAILKKAKKLLKKLVNP) and TP2-6 (KKCIAKAILKKAKKLLKDLVNP) possess a net positive charge of +9 and +7, respectively, with amphipathic characteristics (Supplementary Figures S1–S4) [7]. Both TP2-5 and TP2-6 are nonhaemolytic and noncytotoxic and have significant antimicrobial activity [7]. Hence, we extended our study to develop a potential anti-*A. baumannii* peptide for future application.

### 2.2. Antimicrobial Activity of the Peptides in Normal Media, Serum and Lung Surfactant

TP2-5 and TP2-6 showed significant antimicrobial activity against *A. baumannii* bacterial species in normal media [7]. Therefore, we extended our study to assess their stability in the presence of human serum and lung surfactant. Human serum and lung surfactant were used to imitate the physiological environment. This experiment helped to clarify the extended stability of the peptides by assessing whether they retained their activity in the presence of these substances. TP2-5 and TP2-6 were both significantly active against the wild-type variant of *A. baumannii* in the presence of 50% human serum and 5% lung surfactant (Table 1). Both peptides were active against 12 MDR *A. baumannii* bacterial species (Supplementary Table S1). Additionally, TP2-5 showed significant activity against MDR *A. baumannii* (14B0091) in the presence of 50% human serum (Table 1). However, the presence of human serum markedly reduced the antimicrobial potential of TP2-6. The MIC and MBC values were still 2-fold lower than those of the control peptide (LL-37) and the antibiotic control (meropenem). Human serum and lung surfactants are complex mixtures of multiple enzymes and factors that can degrade or reduce the therapeutic potencies of poly-L peptides [5,6]. Unlike many naturally occurring peptides, the pair of de novo peptides retained their activities when compared with the peptide control (LL-37). However, LL-37 showed lower potency against the MDR-strains in media alone. Apart from enhanced stability, the therapeutic potencies of the peptides against MDR strains are noteworthy (Table 1). Therefore, the current pair of peptides can be further tested and explored for their possible therapeutic applications against MDR infections.

### 2.3. Cytotoxicity Potential of the Peptides

**Figure 1:**
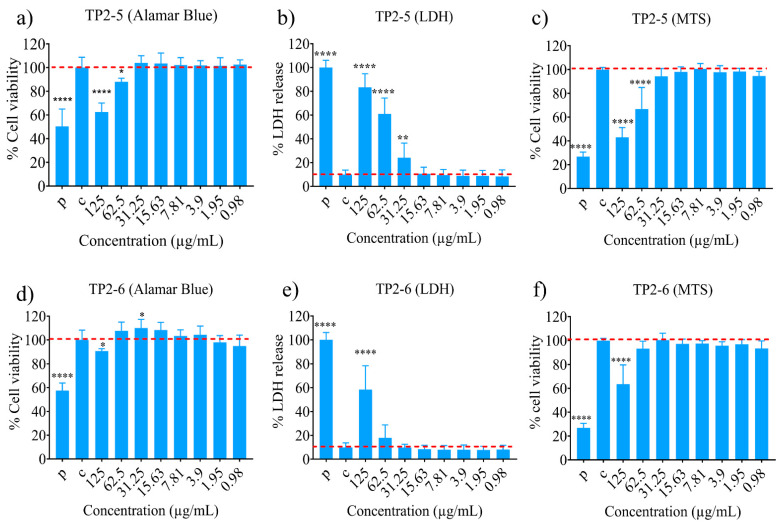
Cytotoxicity potential of the peptides. Cytotoxicity potential of TP2-5 (**a**) Alamar blue, (**b**) LDH and (**c**) MTS assay and of TP2-6 (**d**) Alamar blue, (**e**) LDH and (**f**) MTS assay against human skin fibroblast (CCD966SK) cell lines. The experiments were performed twice in triplicate. The statistical analysis was performed by one-way ANOVA (Dunnett’s test) (* *p* < 0.05, ** *p* < 0.005, **** *p* < 0.0001).

TP2-5 and TP2-6 showed negligible cytotoxicity against human skin fibroblast (CCD966SK) cell lines. The peptides showed relatively low toxicity even at high concentrations (Figure 1a–f). We can assume their safety profiles are acceptable, as the peptides had MIC and MBC values as low as 3.125 to 6.25 µg/mL (Table 1). Low cytotoxicity is an important parameter for any candidate peptide for continued in vivo study. This feature enhances the therapeutic index and is a favorable indicator of safety for a given set of test molecules. For our peptides, the MIC and MBC values of TP2-5 and TP2-6 were between 3.125 µg/mL and 6.25 µg/mL in the presence of human and lung surfactant, whereas toxicity was only observed at a higher dose of 62.5 µg/mL for TP2-5, as assayed by % LDH release. The alamar blue and MTS assays showed notable toxicity at a higher dose of 125 µg/mL TP2-5. TP2-6 showed notable toxicity at 125 µg/mL when measured by LDH release. The MTS and alamar assay profiles for TP2-6 were relatively better than those of TP2-5. These results demonstrate that the MIC and MBC doses of TP2-5 and TP2-6 are significantly lower than the toxic doses. Hence, the current pair of peptides may be safe and effective, making them suitable for further trials.

### 2.4. Haemolysis, Bacterial Killing Kinetics and Induced Resistance Assay

**Figure 2:**
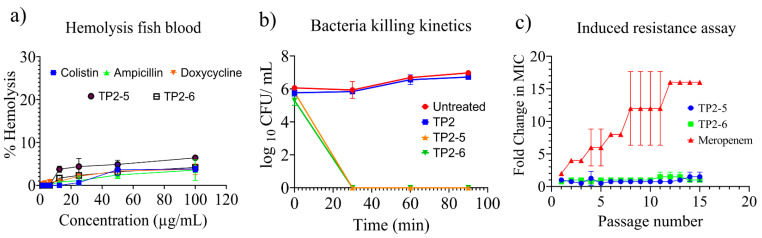
Haemolysis, bacterial killing kinetics and induced resistance assay. (**a**) Haemolysis activity of the peptides and control antibiotics against tilapia fish blood. (**b**) Bacterial killing kinetics of the peptides against *A. baumannii* 10591 bacterial species. (**c**) Induced resistance assay of the peptides and meropenem against *A. baumannii* 10591 for a continuous passage of 15 cycles. All experiments shown here were performed in three replicates in a set of two independent experiments.

The peptides were assessed for their haemolytic potential. Both peptides showed negligible toxicity even at the highest concentration of 100 µg/mL (Figure 2a). Based on the haemolysis data, these peptides were found to be nontoxic, which is an important characteristic for continuation in the drug discovery pipeline. The antimicrobial activity of the peptides was significantly active against both wild-type and resistant variants of *A. baumannii*. To estimate the duration for bacterial clearance, we determined the bacterial killing kinetics of TP2-5 or TP2-6. Both peptides showed 100% bacterial killing against *A. baumannii* 10591 within 30 min (Figure 2b) at their respective MIC/MBC values (Table 1). Antimicrobial agents tend to show reduced activity upon prolonged exposure to microbes at low concentrations. We performed a serial passage assay of TP2-5, TP2-6 and meropenem against *A. baumannii* (10591) for a repetition of 15 cycles (Figure 2c). TP2-5 and TP2-6 showed negligible increases in their MIC values. However, meropenem showed a significant increase in its MIC, which was up to 16-fold higher than the initial MIC value. These results show that TP2-5 and TP2-6 are less prone to induce resistance, whereas clinical antibiotics can rapidly lose their antimicrobial activity upon prolonged exposure to bacterial species.

### 2.5. Antimicrobial Activity of the Peptides in the Presence of Variable Temperature and Physiological Ions

The antimicrobial activity of the peptides was also tested after incubation at high temperatures. The two peptides were incubated at 40, 60, 80 and 100 °C for 1 h prior to the assay. Both peptides showed significant antimicrobial activity even after incubation at 100 °C (Table 2), demonstrating their temperature stability. Meropenem also showed significant stability up to 80 °C; however, it lost activity after the 100 °C incubation (Table 2). TP2-5 and TP2-6 were tested for their activity in the presence of physiological ions and glucose under hyperglycaemic conditions. TP2-5 showed significant activity in the presence of physiological ions and glucose (Table 3). TP2-6 also showed notable activity in the presence of salts; however, it lost significant activity in the presence of CaCl_2_. AMPs tend to lose potency in the presence of ions, compromising their clinical efficacy. The results of the current study showed that the two peptides retained their activity in the presence of physiological ions, an important criterion for clinical application.

### 2.6. FIC Index of the Peptides and Antibiotics

The FIC index was calculated to assess potential synergistic and antagonistic antimicrobial activities of the two peptides with meropenem (Table 4). TP2-5 showed additive activity with meropenem against most of the MDR *A. baumannii* bacterial species, except *A. baumannii* 2998. The FIC index was also determined for the combination of TP2-6 and meropenem against MDR *A. baumannii* bacterial species (Table 5). The combination of TP2-6 and meropenem showed additive activity in most cases, except for *A. baumannii* 2982. Based on this assay, we confirmed the synergistic potential of the peptides and meropenem against multidrug-resistant *A. baumannii* bacterial species. Of note, synergistic activity can allow for the reuse of old antibiotics that were discontinued due to reduced therapeutic potential. Such repurposing of older drugs may be a faster way to develop new therapeutic options due to their well-known clinical effects. This strategy may lead to novel multiple-drug therapy (MDT) combinations that are effective against a variety of life-threatening infections. MDT is a common approach in the application of antibiotics and enzymes. Additionally, clinical studies with natural and synthetic compounds, peptides, and antibody-drug conjugates may performed to identify new therapeutic entities [1]. Hence, the synergistic activities of AMPs with antibiotics could lead to the development of new antimicrobial therapy options.

### 2.7. Antibiofilm Activity of the Peptides

**Figure 3:**
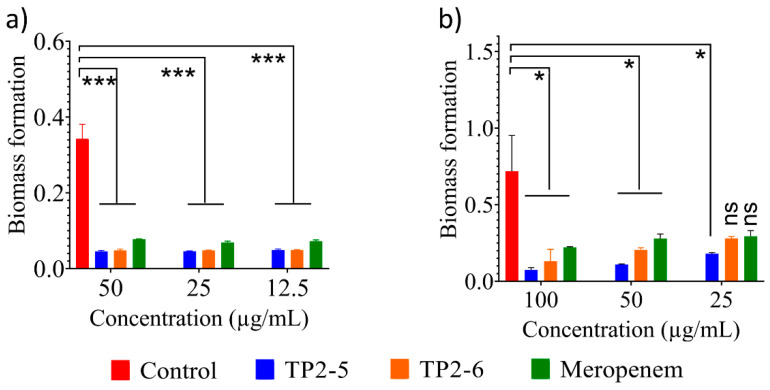
Antibiofilm activity of the peptides. (**a**) Biofilm inhibition and (**b**) rupture induced by meropenem and TP2-5 and TP2-6 at different concentrations against *A. baumannii* 10591. All data are reported as the mean ± SD (*n* = 3 per group) from two independent trials. Statistically significant differences compared to the control (vehicle) were calculated by one-way ANOVA and Dunnett’s test (* *p* < 0.05; *** *p* < 0.001; ns, not significant).

Bacterial biofilms are the most resistant form of bacterial aggregates and are responsible for approximately 80% of bacterial infections [10]. Therefore, we tested the antibiofilm potentials of TP2-5 and TP2-6. Both peptides showed significant biofilm inhibition against *A. baumannii* at the low concentration of 12.5 µg/mL (Figure 3a). TP2-5 and TP2-6 showed significant biofilm rupture at all tested concentrations (Figure 3b). TP2-6 and meropenem showed nonsignificant biofilm rupture at the lowest concentration (25 µg/mL). Biofilms are mostly untreatable with current antibiotics. Therefore, potential antibiofilm molecules are of significant interest to researchers [8]. Given that our pair of AMPs showed low cytotoxic and haemolytic activities, they can be further tested as potential antibiofilm molecules for clinical applications.

### 2.8. Membranolytic Activity of the Peptides

**Figure 4:**
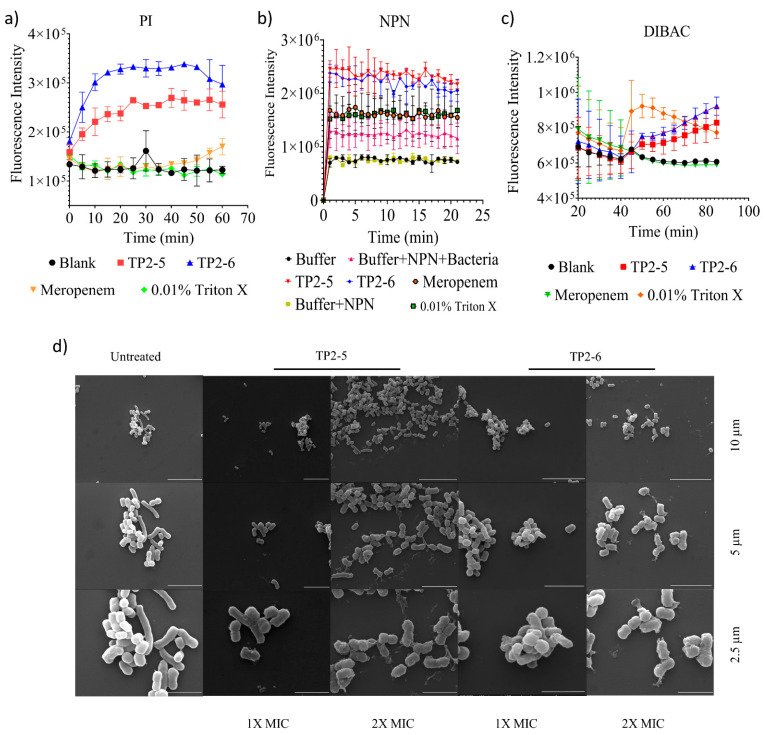
Membranolytic activity of the peptides. (**a**) *A. baumannii* 10591 membrane lysis (propidium iodide-PI) caused by TP2-5 and TP2-6. (**b**) *A. baumannii* 10591 outer membrane lysis (NPN dye) caused by TP2-5 and TP2-6 at 25 µg/mL. (**c**) *A. baumannii* 10591 membrane depolarization (DIBAC4 dye) caused by TP2-5 and TP2-6 at 25 µg/mL. (**d**) SEM analysis of 1x and 2x MIC peptide-treated and untreated *A. baumannii* 10591. Scale bars are 2.5 µm, 5 µm and 10 µm. PI, NPN and DIBAC4-3 data are reported as the mean ± SD (*n* = 3 per group) from two independent trials. Statistically significant differences compared to the control (vehicle) were calculated by one-way ANOVA and Dunnett’s test.

AMPs are known to be membranolytic [9]; therefore, we tested the membranolytic activity of the peptides using propidium iodide reagent. The peptide-treated bacterial species showed a significant increase in fluorescence intensity compared with untreated controls (Figure 4a). Both peptides led to notable outer membrane rupture in the presence of NPN dye (Figure 4b) compared with untreated groups. The peptides were also assessed for their membrane depolarization potential using DIBAC4-3 dye. TP2-5 and TP2-6 showed significant membrane depolarization activity (Figure 4c). To infer the membrane-active nature of the peptides, microscopic analyses of peptide-treated membranes and untreated controls were performed (Figure 4d). The peptide-treated bacterial species showed deformed membrane architecture, whereas untreated controls showed intact membranes. Based on these studies, we can infer that the peptides primarily act through membranolytic activity.

## 3. Materials and Methods

### 3.1. Peptide Synthesis and Characterization

The peptides were synthesized using F-moc chemistry (GL Biochem, Shanghai, China) [11]. In brief, peptides were synthesized on a solid support (Rink amide resin) and then removed from the resin by incubating with a cleavage mixture [trifluoroacetic acid (TFA): ethanedithiol: m-cresol: thioanisole :: 20: 1: 2: 2] for 12–16 h. Later, the cleaved peptides were precipitated in ice-cold ether, followed by repeated washing. The peptides were then purified by reverse-phase high-performance liquid chromatography (RP-HPLC). A gradient was run using Solvent A (100 acetonitrile with 0.1% TFA) and Solvent B (water with 0.1% TFA) was performed at 220 nm with a flow rate of 1 mL/min. Samples were eluted using Sinochrom ODS BP-5 column over a period of 30 min. The molecular masses of the purified peptides (with purity grades of >95%) were verified by electrospray ionization mass spectrometry (ESI-MS). Samples were dissolved in a 50% mixture of Solvent A and B, followed by sample detection with a flowrate (nebulizer gas flow) of 1.5 mL/min (detector −1.5 Kv).

### 3.2. Antimicrobial Assay and Bacterial Killing Kinetics

Bacterial cells *Acinetobacter baumannii* 10591 and 14B0091 were obtained from BCRC Taiwan. All the MDR strains were obtained from the hospital as gifts. The antimicrobial assay was performed as per the standard protocol; 50 µL of 10^6^ CFU/mL of mid-log phase bacterial suspension was treated with 50 µL of candidate peptide and meropenem (Sigma: PHR1772) incubated at 37 °C for 960~1200 min. The clear wells corresponding to the lowest concentrations are reported as the minimum inhibitory concentration (MIC). Suspensions from the clear wells were plated on Mueller–Hinton agar (MHA) plates and incubated at 37 °C overnight [6]. The lowest concentration corresponding to no growth was recorded as the minimum bactericidal concentration (MBC). To detect the effect of human serum (H3667, Sigma Aldrich, St. Louis, MO, USA) on the peptides, the media was replaced with 50% human serum-containing media. Similarly, to detect the effect of lung surfactant (SURVANTA^®^, beractant, AbbVie Inc., Lake Bluff, IL, USA (25 mg/mL)) on the antimicrobial activity of the peptide, the normal media was replaced with 5% lung surfactant-containing media [6]. To analyse the bacterial killing kinetics, after the addition of the peptide to the bacterial suspension, 10 µL of broth was diluted 1000 times, plated at 30 min intervals, and allowed to incubate for 90 min. The bacterial load is expressed as CFU/mL as a function of time [12].

### 3.3. Fish Blood Haemolysis

Fresh Nile tilapia blood was collected and washed three times in normal saline by centrifuging at 800× *g*. Then, 50 µL of 2% blood suspension was treated with 50 µL of test peptide for 1 h at room temperature. The incubated mixture was then centrifuged, and hemoglobin release was recorded using a spectroscope at 570 nm [13].

### 3.4. Cytotoxicity Assay

Cultures of 8 × 10^3^ cells/100 µL (human skin fibroblast (CCD966SK) were incubated for 24 h in the presence of 5% CO2, followed by peptide treatment for 24 h. Peptide-treated cell were assessed for cytotoxicity using an LDH cytotoxicity detection kit [14], Alamar blue cell viability reagent [15] and a non-radioactive cell proliferation assay (MTS) [16]. For the control for the study, 0.1% Triton X (P) and normal media (C) was used.

### 3.5. Antibiofilm Assay

For the biofilm inhibition assay, 106 bacterial cells were treated with peptide at various concentrations in MHB media containing 0.2% glucose for 24 h at 37 °C in 96-well plates. The wells were then washed with PBS, treated with 100% methanol for 15 min, and the washes were discarded. Crystal violet solution (0.02%) was added to the dry plates and incubated for 30 min. The wells were then washed and treated with 33% acetic acid, followed by detection at 595 nm. For the biofilm rupture assay, 106 bacterial cells were grown overnight. The residual liquid was discarded, followed by washing with PBS. Next, 100 µL of peptide at different concentrations was added as estimated according to the biofilm inhibition assay [17,18].

### 3.6. Peptide Sensitivity to Temperature

Here, 50 µL of 10^6^ CFU/mL bacterial cells were treated with peptide previously incubated at 40, 60, 80, and 100 °C for 1 h [19,20]. The peptide activity was reported as per the standard MIC and MBC protocols.

### 3.7. Induced Resistance Assay

A total of 50 µL of 10^6^ CFU/mL bacterial cells were treated with different concentrations of peptide. Bacterial cells from subinhibitory wells were subcultured by passaging for 15 cycles, followed by testing their MIC values. The fold change in MIC was reported in comparison with starting MIC values [21].

### 3.8. Fractional Inhibitory Concentration (FIC) Index Assay

A 2-fold dilution of peptide in 50 µL of media was performed along the vertical axis of a 96-well plate. Similarly, 50 µL of the next drug was diluted 2-fold along the horizontal axis. Next, 50 µL of 106 cells was added, and the standard MIC protocol was performed [22]. The FIC index was calculated according to the following formula:

FICI = FIC A + FIC B

where FIC A is MIC A & B combined/MIC A and FIC B is MIC A & B combined/MIC A.

### 3.9. Membrane Lysis Assay

A total of 108 bacterial cells were dispersed in MHB medium containing 20 µg/mL propidium iodide (PI). Then, 90 µL of the bacterial suspension was mixed with 10 µL of peptide in a 96-well black plate. Membrane rupture was recorded (excitation = 584, emission = 620) in the peptide-treated and untreated (blank) bacterial cells [23].

### 3.10. Outer Membrane Permeabilization Assay

A total of 108 bacterial cells were suspended in PBS containing 25 mM glucose, followed by incubation at 37 °C for 15 min. Post incubation, N-phenyl-1-naphthyl amine (NPN) was added to a final concentration of 10 µM. A 90 µL aliquot of the bacterial suspension was recorded as the blank (350 nm excitation, 420 emission) for 40 min. Next, 10 µL of peptide was added, and spectra were recorded for 20 min [24].

### 3.11. Membrane Depolarization Assay

A total of 108 bacterial cells were washed and resuspended in PBS containing 25 mM glucose. Bis-(1,3-dibarbituric acid)-trimethine oxanol (DIBAC) was then added to a final concentration of 500 nM. Ninety microlitres of the bacterial blank was recorded (excitation 490, emission 516) for 40 min, followed by peptide treatment (10 µL), and the spectra were recorded for 40 min [25].

### 3.12. Field Emission Scanning Electron Microscopy

The bacterial cells were treated at the respective MICs of the peptides. The cells were then washed and resuspended in 4% glutaraldehyde in PBS and incubated at 4 °C for 30 min. The cells were then fixed on glass slides for 15 min. The slides were subsequently washed with ethanol (20% to 100%). Dry slides were gold-coated, and images were recorded using an electron microscope [13].

## 4. Conclusions

AMPs and their derivatives are widely considered to be potential alternatives to existing antibiotics [5]. Several modified peptides, such as peptide–drug conjugates, are currently being tested in clinical trials, but only a few AMPs are in clinical use [1]. Importantly, AMPs show significant potencies against a vast array of multidrug-resistant pathogens in laboratory studies. However, their efficacies are often compromised in the presence of physiological factors, which is a major obstacle to their clinical application [5]. To overcome this and other obstacles, systematic modification of peptide sequences or peptidomimetic approach can be a possible route to develop new AMPs with enhanced activities and stabilities in vivo [5]. In the current study, we sought to design and develop potential AMPs against MDR *Acinetobacter baumannii* bacterial species. We successfully developed a pair of stable peptides that show significant activities in the presence of 50% human serum and 5% lung surfactants. This stability was due to targeted changes in the peptide sequences, which resulted in the development of new AMPs. Both of the peptides possess relatively higher densities of net positive charge with optimal amphipathicity, as compared to the parental TP2 peptide molecule. Most importantly, the modified peptides showed significant activity against MDR bacterial species, with low toxicity to human cells and minimal hemolytic activity. Additionally, the peptides showed significant antibiofilm activity. Both TP2-5 and TP2-6 acted synergistically with meropenem, and the designed peptides retained their activities in the presence of physiological ions and elevated temperature. Additionally, the peptides showed lower degrees of induced resistance than meropenem. The mechanism of bactericidal activity for the peptides was via lysis of the bacterial membrane. Based on our collective results, the peptides show significant potential for further study. Hence, these two peptides should be evaluated in further trials and studies, which may lead to the development of promising drug candidates to fight antimicrobial resistance.

## Figures and Tables

**Table 1:** Minimal inhibitory concentration (MIC) and minimal bactericidal concentration (MBC) of TP2-5 and TP2-6 against wild-type *A. baumannii* and MDR *A. baumannii* species in the presence of human serum and lung surfactant. All experiments were performed in triplicate in two independent experiments. Data are expressed in µg/mL.

Peptides/Drugs		TP2-5 (µg/mL)		TP2-6 (µg/mL)		LL-37 (µg/mL)		Meropenem (µg/mL)	
Bacterial Strain		MIC	MBC	MIC	MBC	MIC	MBC	MIC	MBC
Wild strain	*A. baumannii* 10591 ^a^	3.125	3.125	3.125	3.125	>100	>100	1.56	1.56
	*A. baumannii* 10591 ^b^	3.125	3.125	3.125	3.125	>100	>100	1.56	1.56
MDR strain	14B0091	3.125	3.125	3.125–6.25	3.125–6.25	12.5–25	12.5–25	>100	>100
	2088	3.125	3.125	3.125	3.125	25	25	>100	>100
	921	3.125	3.125	6.25	6.25	12.5	12.5	100	>100
	1019	3.125	3.125	6.25–12.5	6.25–12.5	12.5	12.5	25	25
	1033	3.125	3.125	6.25	6.25	25	25	50	100
	1607	1.56	1.56	6.25	6..25	12.5	12.5	50	50
	1702	3.125	3.125	6.25	6.25	12.5	12.5	12.5	12.5
	2962	3.125–6.25	6.25	3.125–6.25	6.25	25	25	50	50
	2982	3.125	3.125–6.25	3.125	3.125	25	25–50	>100	>100
	2997	1.56–3.125	1.56–3.125	6.25	6.25	12.5	12.5	50–100	50–100
	2998	1.56–3.125	1.56–3.125	3.125–6.25	3.125–6.25	25	50	>100	>100
	3618	3.125	3.125	6.25	6.25	12.5	12.5	50–100	50–100
	14B0091 ^c^	12.5	12.5	50	50	>100	>100	>100	>100

^a^, ^c^ media containing 50% human serum, ^b^ media containing 5% lung surfactant.

**Table 2:** Temperature-dependent antimicrobial activity of meropenem, TP2-5 and TP2-6 against *A. baumannii*. The data were derived from triplicate samples in two individual experiments. Data are expressed in µg/mL.

*A. baumannii*	TP2-5 (µg/mL)		TP2-6 (µg/mL)		Meropenem (µg/mL)	
	MIC	MBC	MIC	MBC	MIC	MBC
40 °C	6.25	6.25	12.5	12.5	1.56	1.56
60 °C	6.25	6.25	12.5	12.5	1.56	1.56
80 °C	6.25	6.25	12.5	12.5	3.125	3.125
100 °C	6.25	6.25	12.5	12.5	50	50

**Table 3:** Antimicrobial activity of meropenem, TP2-5 and TP2-6 against *A. baumannii* 10591 in the presence of physiological ions and glucose. The data were derived from triplicate samples in two individual experiments. Data are expressed in µg/mL.

*A. baumannii*	TP2-5 (µg/mL)		TP2-6 (µg/mL)	
	MIC	MBC	MIC	MBC
CaCl_2_	12.5	12.5	50	50
MgCl_2_	3.125	3.125	12.5	12.5
NH_4_Cl	1.56	1.56	3.125	3.125
KCl	3.125	3.125	3.125	3.125
NaCl	6.25	6.25	12.5	12.5
Glucose	1.56	6.25	6.25	6.25

**Table 4:** MIC values and synergistic activities of TP2-5 in combination with meropenem. MICs for drug alone and in combination were determined in two experiments performed in triplicate. Data are expressed in µg/mL.

Bacteria	MIC Alone (µg/mL)		MIC in Combination (µg/mL)		FICI	Interaction
	TP2-5	Meropenem	TP2-5	Meropenem		
14B001	3.125	400	1.56	200	1	Additive
2088	3.125	200	1.56	100	1	Additive
921	3.125	100	0.0976	100	0.53	Additive
1019	3.125	25	0.78	12.5	0.75	Additive
1033	3.125	50	0.0976	50	1.03	Indifferent
1607	1.56	25	0.0488	25	0.53	Additive
1702	3.125	12.5	0.0976	12.5	0.53	Additive
2962	6.25	50	(0.19	25	0.53	Additive
2982	3.125	100	1.56	100	1.5	Indifferent
2997	3.125	100	1.56	50	1	Additive
2998	3.125	200	0.096	200	1.03125	Indifferent
3618	3.125	100	0.096	50	0.28125	Synergistic

**Table 5:** MIC values and synergistic activities of TP2-6 in combination with meropenem. MICs for drug alone and in combination were determined in two experiments performed in triplicate. Data are expressed in µg/mL.

Bacteria	MIC Alone (µg/mL)		MIC in Combination (µg/mL)		FICI	Interaction
	TP2-6	Meropenem	TP2-6	Meropenem		
14B001	6.25	400	3.125	200	1	Additive
2088	3.125	200	1.56	100	1	Additive
921	6.25	100	0.39	50	0.5625	Additive
1019	12.5	25	3.125	12.5	0.375	Synergistic
1033	6.25	50	3.125	25	1	Additive
1607	6.25	25	1.56	25	0.75	Additive
1702	6.25	12.5	3.125	1.56	0.625	Additive
2962	6.25	50	0.78	25	0.625	Additive
2982	3.125	100	0.097	100	1.03125	Indifferent
2997	6.25	100	1.56/3.125	50/25	0.75	Additive
2998	6.25	200	3.125	100	1	Additive
3618	6.25	100	3.125	25	0.625	Additive

## Data Availability

The data presented in this study are available on request from the corresponding author.

## Supplementary Materials

The following are available online at https://www.mdpi.com/article/10.3390/ijms23042191/s1.
