# Supplementary material for: Development of Bactericidal Peptides against Multidrug-Resistant Acinetobacter baumannii with Enhanced Stability and Low Toxicity
Source: Int J Mol Sci. 2022 Feb 16;23(4):2191. doi: 10.3390/ijms23042191 (PMC8875074; doi:10.3390/ijms23042191)
Supplement: Supplementary file 1 [file ijms-23-02191-s001.zip › ijms-1586270-SI.pdf]

Development of Bactericidal Peptides against Multidrug Resistant Acinetobacter Baumannii with Enhanced Stability and Low Toxicity

Author and affiliation

Prakash Kishore Hazam [1,†], Chin-Cheng Cheng [2,†], Chu-Yi Hsieh [1], Wen-Chun Lin [1], Po-Hsien Hsu [2], Te-Li Chen [3,4], Yi-Tzu Lee [5,6] and Jyh-Yih Chen [1,7,*]

[1] Marine Research Station, Institute of Cellular and Organismic Biology, Academia Sinica, 23-10 Dahuen Rd., Jiaushi, Ilan 262, Taiwan; prakashkishor.hazam@gmail.com (P.K.H.); joy.cde49@gmail.com (C.-Y.H.); hagirl19@gmail.com (W.-C.L.)

[2] Institute of Fisheries Science, National Taiwan University, 1 Roosevelt Road, Sec. 4, Taipei 106, Taiwan; eddie040886@gmail.com (C.-C.C.); ericchu102001@gmail.com (P.-H.H.)

[3] Graduate Institute of Life Sciences, National Defense Medical Center, 114, Taipei, Taiwan; tecklayyy@gmail.com

[4] Institute of Clinical Medicine, School of Medicine, National Yang-Ming University, 112-21,Taipei, Taiwan

[5] Department of Emergency Medicine, Taipei Veterans General Hospital, No.201, Section 2, Shipai Road, Taipei 11217, Taiwan; s851009@yahoo.com.tw

[6] Faculty of Medicine, School of Medicine, National Yang-Ming University, No.155, Sec.2, Linong Street, Taipei 11221, Taiwan

[7] The iEGG and Animal Biotechnology Center and the Rong Hsing Research Center for Translational Medicine, National Chung Hsing University, Taichung 402, Taiwan

* Correspondence: zoocjy@gate.sinica.edu.tw

† These authors contributed equally.

Supplementary information

Supplementary Table S1: Details of antibiotic resistance of the selected MDR *Acinetobacter baumannii* bacterial species obtained as a gift sample from the hospital.

S. no	Bacteria strain number	Multiplex PCR	OXA51-like	OXA58-like	Amikacin	Ampicillin-sulbactam	Cefepime	Ceftazidime	Ciprofloxacin	Colistin	DOR	Gentamicin	Imipenem	Minocycline	Meropenem	Piperacillin-Tazobactam	Sulbactam	Tigecycline
1	921	Ab	+	+	>2048	64/32		128	128	1		>1024	32		64		16	2
2	1019	Ab	+	+	>2048	64/32		256	64	1		>1024	32		16	1024/4	32	1
3	1033	Ab	+	+	>2048		32	256	64	1	32	>1024	32	4	64	256/4	16	2
4	1607	Ab	+	+	>32				>2	1		>8	32		32	>64	16	
5	1702	Ab	+	+	>2048		32	512	256	1		>1024	32		16	2048/4	8	0.5
6	2962	Ab	+	+	>128		>32	>32	64	2	128	>32	16	4	16			
7	2982	Ab	+	+	>2048	128/64	128	128	128	4		>4096	64		128	4096/4		
8	2997	Ab	+	+	>2048	128/64	128	512	>8	4	32	>4096	64	2	64	1024/4		
9	2998	Ab	+	+	>2048	128/64	256	128	128	4		>4096	64		128	4096/4	32	2
10	3618	Ab	+	+	>2048	128/64	64	512	256	4	64	>1024	32	4	64	1024/4		

Note: *A. baumannii 14B0091 BCRC(https://catalog.bcrc.firdi.org.tw/BcrcContent?bid=14B0091&rowid=1) resistance to Ampicillin, Cefazolin ,Cefoxitin, Ceftazidime, Ceftriaxone, Ciprofloxacin, Penicillin, Tetracycline, Imipenem. 2088 is resistant to meropenem and doxycycline.*

TP2-5 HPLC

Sample Description:

Structure:KP-22

Number: 0200046

Lot No:P200330-SY786185

Column: 4.6mm*250mm, Inertsil ODS-SP

Solvent A: 0.1%Trifluoroacetic in 100% Acetonirile

Solvent B: 0.1%Trifluoroacetic in 100% Water

Gradient:	A	B
0.01min	15%	85%
25.00min	40%	60%
25.01min	100%	0%
30.00min	Stop	

Flow rate:1.0ml/min

Wavelength:220nm

Volume:10μl

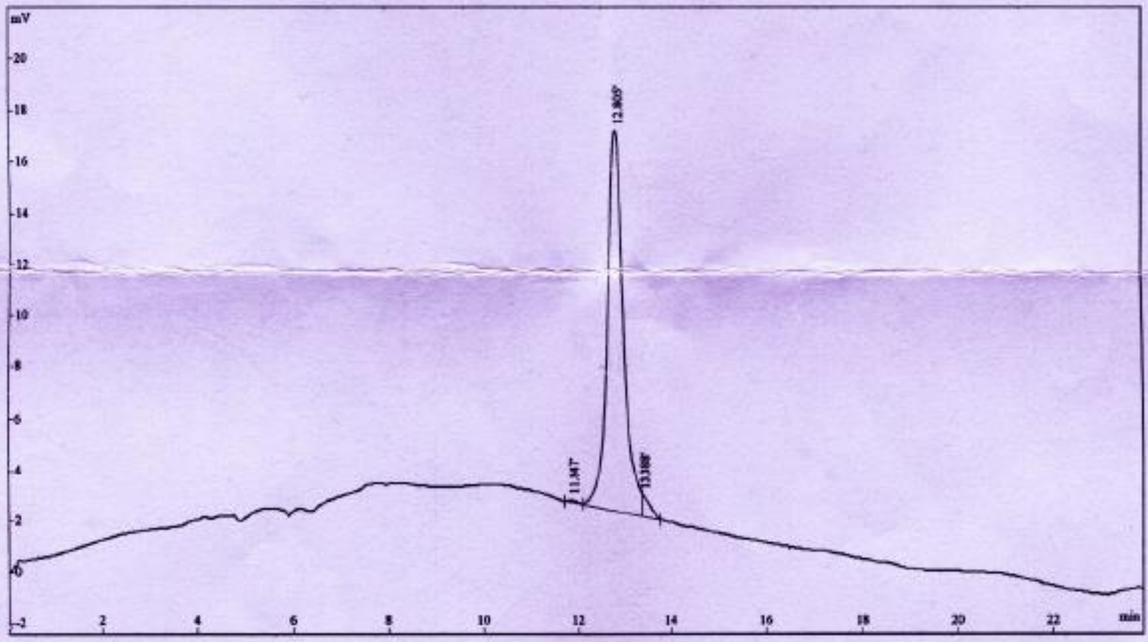

Rank	Time	Conc.	Area	Height
1	11.847	0.4914	1736	117
2	12.805	97.35	344015	14854
3	13.388	2.155	7614	780
Total		100	353365	15751

Supplementary Figure S1: HPLC profile of TP2-5

TP2-5 Mass spectrometry

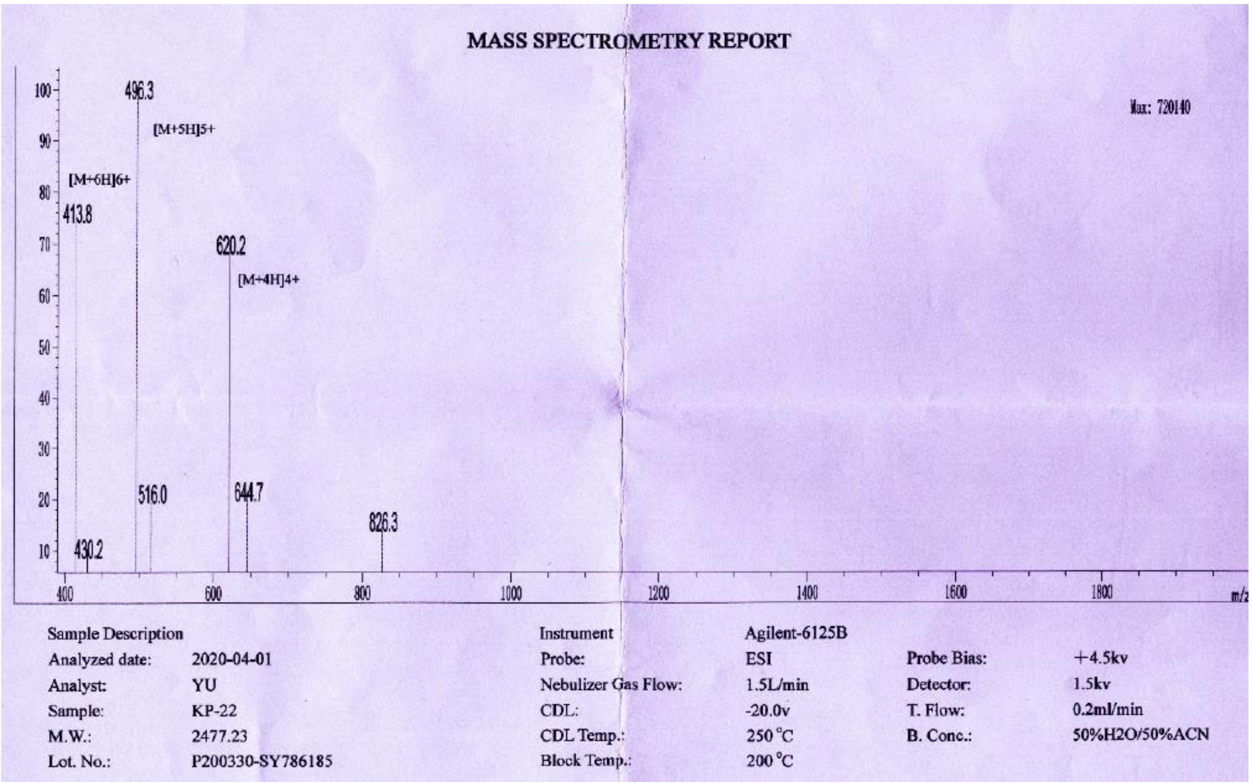

Supplementary Figure S2: Mass spectrometry profile of TP2-5

TP2-6 HPLC

TP2-6 (KP-22) REPORT

Product Name:KP-22

Instrument No: 0200047

Lot No ;P200929-MX786186

Column :4.6*250mm, kromasil C18-5

Solvent A :0.1%Trifluoroacetic in 100% Acetonitrile

Solvent B :0.1%Trifluoroacetic in 100% Water

Gradient :	A	B
0.01min	30%	70%
25min	55%	45%
25.1min	100%	0%
30min	Stop	

Flow rate :1.0ml/min

Wavelength :220nm

Volume:5ul

File opened: F:786186-30-55.hw, where

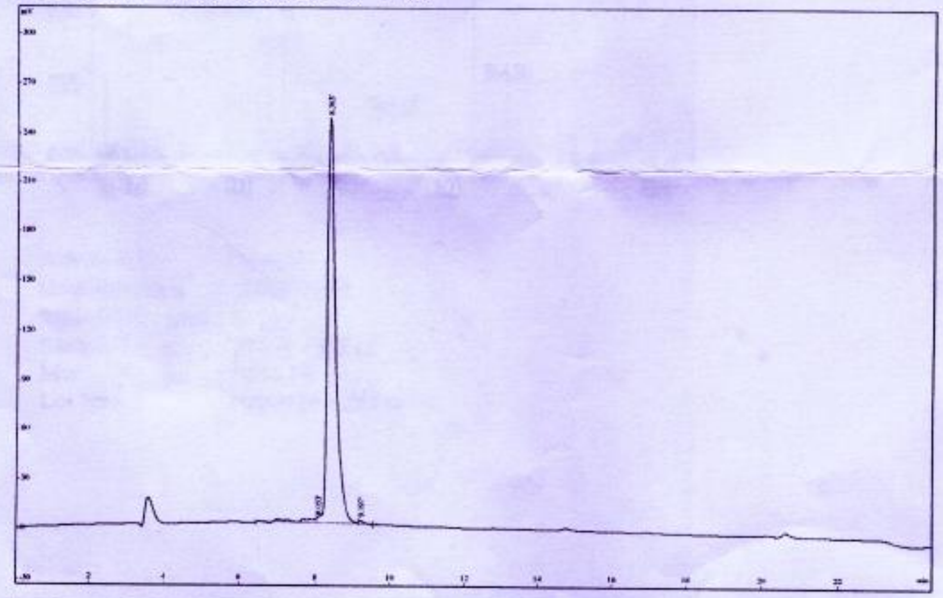

Rank	Time	Conc.	Area	Height
1	8.053	3.198	121326	3112
2	8.363	96.26	3651605	246979
3	9.197	0.5481	20793	2030
Total		100	3793724	252121

Supplementary Figure S3：HPLC profile of TP2-6

TP2-6 Mass spectrometry

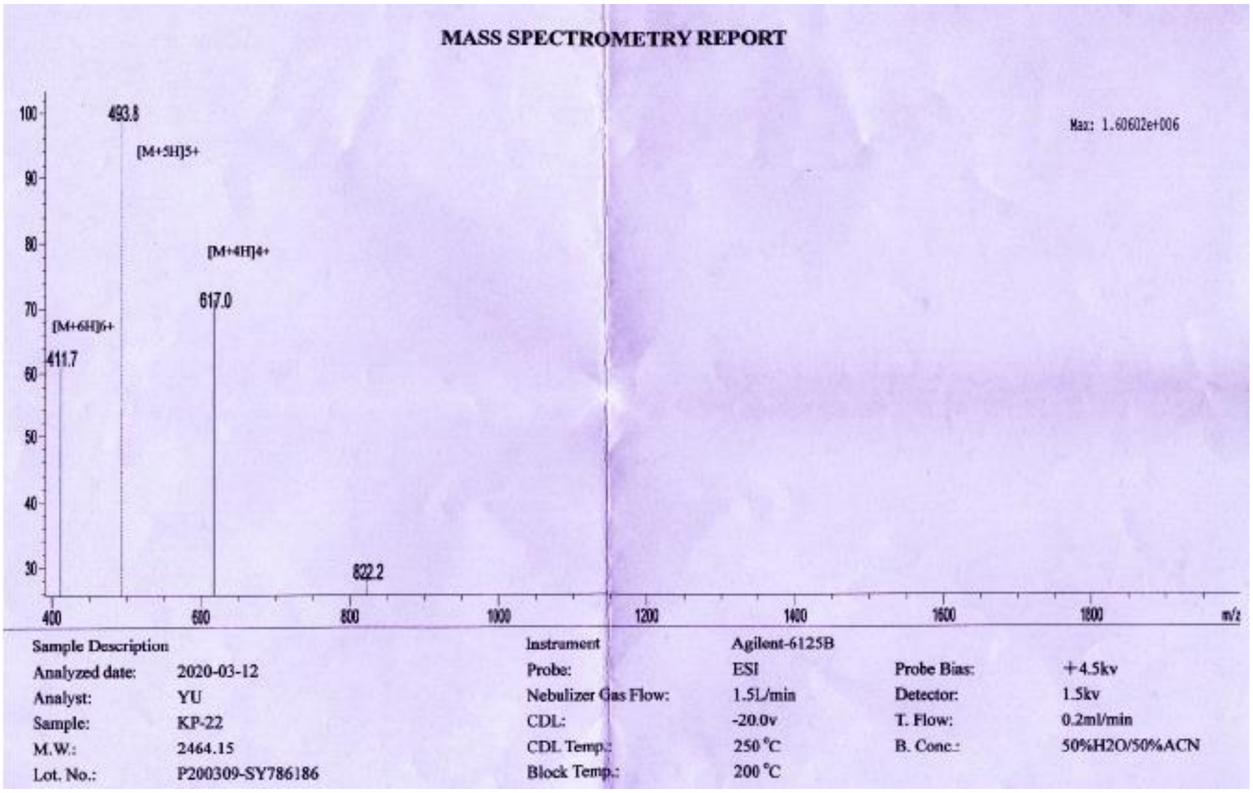

Supplementary Figure S4: Mass spectrometry profile of TP2-6
